# Molecular insights and inhibitory dynamics of flavonoids in targeting Pim-1 kinase for cancer therapy

**DOI:** 10.3389/fphar.2024.1440958

**Published:** 2024-10-07

**Authors:** Hani A. Alhadrami, Ahmed M. Sayed, Hossam M. Hassan, Albaraa H. Alhadrami, Mostafa E. Rateb

**Affiliations:** ^1^ Faculty of Applied Medical Sciences, Department of Medical Laboratory Sciences, King Abdulaziz University, Jeddah, Saudi Arabia; ^2^ King Fahd Medical Research Centre, DNA Forensic Unit, King Abdulaziz University, Jeddah, Saudi Arabia; ^3^ King Abdulaziz University Hospital, Molecular Diagnostics Lab, Jeddah, Saudi Arabia; ^4^ Department of Pharmacognosy, Faculty of Pharmacy, Nahda University, Beni Suef, Egypt; ^5^ Department of Pharmacognosy, Faculty of Pharmacy, Beni-Suef University, Beni Suef, Egypt; ^6^ School of Computing, Engineering & Physical Sciences, University of the West of Scotland, Paisley, United Kingdom

**Keywords:** flavonoids, Pim-1 kinase inhibitors, steered molecular dynamics, computational, targeted cancer therapy, linear regression

## Abstract

Pim-1 kinase, a serine/threonine kinase, is often overexpressed in various cancers, contributing to disease progression and poor prognosis. In this study, we explored the potential of flavonoids as inhibitors of Pim-1 kinase using a combination of molecular docking and steered molecular dynamics (SMD) simulations. Our docking studies revealed two main binding orientations for the flavonoid molecules. The SMD simulations showed that the binding mode with higher pulling forces was linked to stronger inhibitory activity, with a strong positive correlation (*R* ^*2*^ ≈ 0.92) between pulling forces and IC_50_ values. Quercetin stood out as the most potent inhibitor, showing a pulling force of about 820 pN and an IC_(5) 0 of less than 6 µM. Further dynamic simulations indicated that quercetin’s hydroxyl groups at the C3, C-5 and C-7 positions formed stable hydrogen bonds with key residues GLU-121, Leu-44 and Val-126, respectively enhancing its binding stability and effectiveness. Our results emphasized the critical role of the hydroxyl group at the C-3 position, which plays a pivotal function in effectively anchoring these molecules in the active site of Pim-1 kinase. Principal component analysis (PCA) of Pim-1 kinase’s conformational changes revealed that potent inhibitors like quercetin, galangin, and kaempferol significantly restricted the enzyme’s flexibility, suggesting potential inhibitory effect. These findings provide insights into the structural interactions between flavonoids and Pim-1 kinase, offering a foundation for future experimental investigations. However, further studies, including *in vitro* and *in vivo* validation, are necessary to assess the pharmacological relevance and specificity of flavonoids in cancer therapy.

## 1 Introduction

PIM-1 kinase, a proto-oncogene-encoded serine/threonine kinase, plays a crucial role in cancer development and progression by regulating the cell cycle, apoptosis, and transcriptional activation (Chen and Tang, 2019; Nafie et al., 2020). Overexpression of PIM-1 is linked to poor prognosis and therapy resistance in various cancers, including hematological malignancies and solid tumors (Castanet et al., 2023). Specifically, PIM-1 overexpression has been observed in diffuse large B-cell lymphoma (DLBCL), acute myeloid leukemia (AML), and chronic lymphocytic leukemia (CLL), correlating with advanced disease stages and reduced survival rates. In solid tumors, high PIM-1 levels are associated with aggressive phenotypes in breast cancer, pancreatic cancer, and head and neck squamous cell carcinoma (Chen and Tang, 2019; Nafie et al., 2020; Castanet et al., 2023).

PIM-1 promotes tumorigenesis by enhancing cell survival and proliferation and modulating key signaling pathways like JAK/STAT and PI3K/Akt (Zhao et al., 2022). Consequently, PIM-1 is a promising target for anticancer therapies aimed at disrupting its oncogenic activity without harming normal cells (Kim et al., 2020a).

In prostate cancer, PIM-1 activation is particularly significant, contributing to tumor progression and aggressiveness, especially in castration-resistant prostate cancer (CRPC) (Heyder et al., 2023). Overexpression in prostate cancer leads to increased cell survival, proliferation, and resistance to apoptosis, exacerbating the disease’s aggressiveness and poor prognosis (Razmazma et al., 2020; Shaban et al., 2023).

The heightened activity of PIM-1 in these cancers is linked to various oncogenic processes, such as promoting cell survival, enhancing cell cycle progression, and inhibiting apoptosis. Its interaction with other molecular pathways, like the JAK/STAT and PI3K/Akt pathways, is critical in driving cancer cell proliferation and survival, making it a key player in tumor growth and resistance to therapy. The prevalence of PIM-1 kinase in these cancers, coupled with its crucial role in disease progression, underscores the importance of targeting PIM-1 for therapeutic interventions in prostate cancer and potentially other hematological malignancies where its expression is dysregulated (Asati et al., 2019). The impact of PIM-1 in prostate cancer underscores its importance in oncology and the ongoing efforts to develop targeted therapies against it (Liu et al., 2020).

In drug discovery, structure-based design techniques are crucial for identifying initial hits, transitioning from hit-to-lead, and optimizing leads (Maia et al., 2020; Aplin et al., 2022; Ibrahim et al., 2022). Molecular docking is a key method, integrating technological advances to address the complexities of ligand-receptor interactions (Kumar and Kumar 2019; Macchiagodena et al., 2020). While recent strategies have improved receptor flexibility accommodation, fully capturing protein conformational shifts during ligand binding remains challenging (Sethi et al., 2019). Predicting ligand binding affinities accurately is still difficult (Acharya et al., 2019; Mishra and Sharma, 2016). Integrative protocols using physics-based simulations have made significant progress, enhancing docking results and providing a more detailed analysis of ligand-protein interactions (Tiwari and Singh, 2022; Sayed et al., 2023; Alasiri et al., 2024). Despite these advancements, using computed binding affinities in early lead discovery and optimization phases remains relatively uncommon.

Steered molecular dynamics (SMD) simulations have become a powerful tool for understanding the energy dynamics of ligand-receptor binding and detailing the sequential formation of complexes (Zhuang et al., 2021). Similar to single-molecule techniques like atomic force microscopy and laser optical tweezers, SMD allows researchers to manipulate molecular structures, exploring their mechanical behaviors in various ways. When combined with non-equilibrium models, SMD can provide quantitative data (Boubeta et al., 2019; Zhang et al., 2020). Although SMD is widely used to study ligand unbinding and ligand-protein interactions, its potential for identifying docking sites and selecting biologically potent compounds for drug discovery is not fully realized. Our research leverages SMD simulations to discover drugs targeting Pim-1 kinase.

**FIGURE 1:**
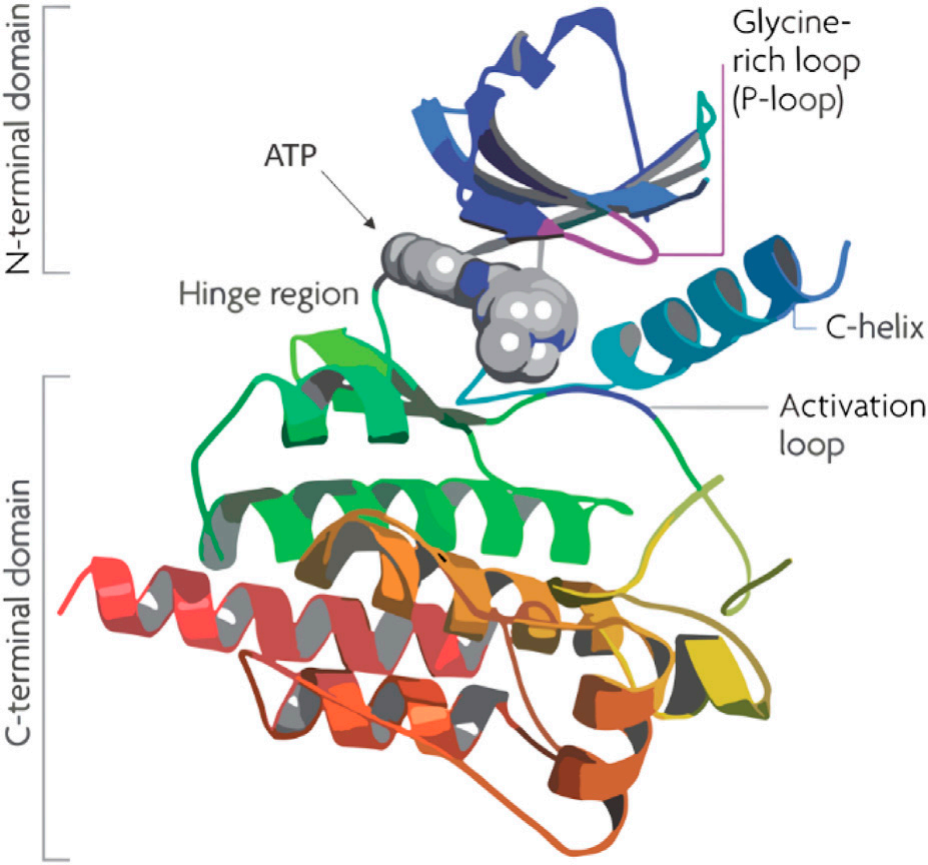
Structure of human Pim-1 kinase.

Flavonoids, a group of naturally occurring polyphenolic compounds found in fruits, vegetables, and other plants, have garnered significant attention in cancer research for their therapeutic potential (Zhao et al., 2019; Kopustinskiene et al., 2020). Quercetin, a well-known flavonoid, stands out for its effectiveness in inhibiting PIM-1 kinase, a crucial enzyme in the progression of cancers such as prostate and breast cancer. Its co-crystallization with PIM-1 kinase has provided detailed insights into its inhibitory mechanism, which occurs mainly at the ATP-binding site of the kinase (Figure 1), thereby blocking its activity. This inhibition can reduce cancer cell growth and promote apoptosis, presenting quercetin as a promising, less toxic alternative for cancer therapy (Holder et al., 2007). By inhibiting Pim-1 kinase, quercetin has been suggested to potentially reduce cancer cell growth and promote apoptosis, though the clinical translation of these effects remains an ongoing challenge (Parker et al., 2014).

Numerous studies have investigated how flavonoids inhibit PIM-1 kinase, aiming to understand their potential to slow down cancer progression (Chao et al., 2015). These studies have used various methods, including quantitative structure-activity relationship (QSAR) analyses and modeling, to identify the most effective flavonoid inhibitors (Lilly et al., 2004). Despite these efforts, there is still a significant gap in applying a rational, structure-based approach to pinpoint the best flavonoid inhibitor. Our study aims to address this gap by leveraging steered molecular dynamics (SMD) simulations to investigate the interaction dynamics between flavonoids and Pim-1 kinase at the molecular level (Zhao et al., 2019). This approach offers a novel perspective on flavonoid binding behavior, with an emphasis on how specific structural features contribute to their inhibitory potential.

**FIGURE 2:**
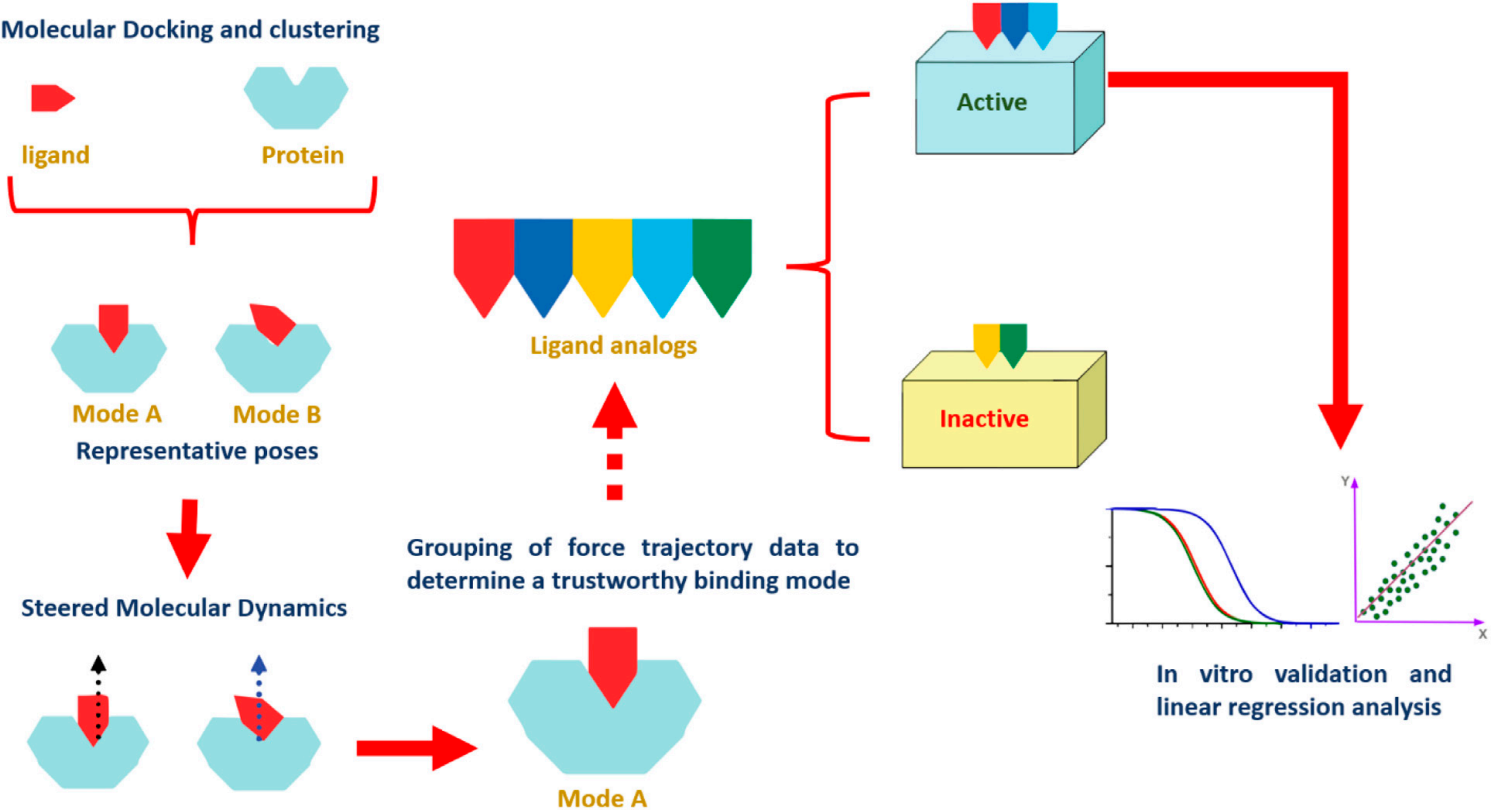
A stepwise illustration of the computational approach applied in the present study.

Our study outlines an integrated computational methodology based on structural analysis to probe molecular interactions and evaluate the binding dynamics of various flavone analogs using *in silico* techniques. Building on the recent identification of flavonoid PIM-1 kinase inhibitors, we combined molecular docking with SMD simulations. This comprehensive approach allowed us to distinguish active flavonoids from inactive ones by analyzing force profiles from SMD simulations related to ligand dissociation. Additionally, this method provided an in-depth understanding of the structure-activity relationship (SAR) among the flavonoids studied. While this study provides a structural framework for understanding flavonoid interactions with Pim-1 kinase, it is essential to recognize that computational findings are inherently limited in predicting pharmacological outcomes. Therefore, further experimental validation, including *in vitro* and *in vivo* studies, is necessary to substantiate the therapeutic potential of these flavonoids in cancer therapy. Our results, however, contribute valuable insights that can inform future investigations and optimization of flavonoid-based inhibitors. The schematic in Figure 2 summarizes the extensive scope of our investigations.

## 2 Materials and methods

### 2.1 Reagents and chemicals

Flavonoids (6-14) were obtained from our in-house natural products library (Alhadrami et al., 2020; Mohamed et al., 2022). Flavonoids one to five were purchased from Sigma Aldrich. All compounds used for the *in vitro* assays were of ≈97% purity.

### 2.2 *In vitro* assay

Enzyme activity was quantified using a luminescence-based kinase assay, which tracks the conversion of ADP to ATP, subsequently producing light via Ultra-Glo™ Luciferase. This luminescence is indicative of ADP levels and thus, enzyme activity. This method is particularly effective for analyzing the influence of various chemicals on different kinases, serving both initial screenings and specificity profiling. The protocol involves preparing dilutions of enzyme, substrate, ATP, and potential inhibitors in kinase buffer, followed by sequential incubation and addition of reagents, with the final step being the measurement of luminescence (El-Hawary et al., 2018; AboulMagd et al., 2020).

### 2.3 Computational study

#### 2.3.1 Docking-based virtual screening

##### 2.3.1.1 Structure generation

Version 3.1.1 of OpenBabel was utilized to transform SMILES notation into three-dimensional models. These models were then subjected to energy reduction using the steepest descent method with the same software. The MMFF94 force field was employed for this energy minimization process (O'Boyle et al., 2011).

##### 2.3.1.2 Ligands preparation

Torsion angles in the gathered molecular structures were identified, and Gasteiger partial charges were computed for each atom using the AutoDock Tools software v.4.2. Structures exhibiting over 32 torsion angles were excluded from the dataset to streamline the analysis (Morris et al., 2009).

##### 2.3.1.3 Protein structure preparation

The Pim-1 kinase structure complexed with quercetin (PDB ID: 4LMU) (Parker et al., 2014), was employed in the docking studies. The structure was refined using PDBfixer software to address any missing components and remove water molecules and heteroatoms. Following this, AutoDock Tools v.4.2 was used to append polar hydrogens and calculate Gasteiger charges for the receptor (Morris et al., 2009; Eastman et al., 2013).

##### 2.3.1.4 Ligand-protein docking

PyRx’s integrated AutoDock Vina software was employed for the molecular docking phase. The docking search’s binding sites were determined according to the enzyme’s bound quercetin. The grid box coordinates were set around the ligand with dimensions appropriate to the enzyme’s active site to ensure only suitably sized molecules were considered. An exhaustiveness level of 24 was maintained to ensure thorough sampling. The resulting docking conformations were then examined and visualized using Pymol software (Seeliger and de Groot, 2010; Dallakyan and Olson, 2015).

#### 2.3.2 Molecular dynamics simulation

Molecular dynamics simulations were conducted using Desmond v. 2.2, employing the OPLS-2005 force field. System Builder was used to prepare protein systems, ensuring correct hydrogen addition and amino acid protonation at pH 7.4 while removing co-crystallized water. Structures were immersed in an orthorhombic TIP3P water box, balanced with 0.15 M Na^+^ and Cl^−^ ions, and subjected to a 20 Å^3^ solvent buffer. Following energy minimization and a 10 ns equilibration, the protein-ligand poses were chosen for simulations, with ligand parameters set by Desmond’s automated OPLS force field application. For simulations using NAMD 3.0, structures were prepared with QwikMD, and compound parameters were derived via the CHARMM27 force field using Ligand Reader and Modeler, ensuring error-free protein-ligand complex readings in VMD for subsequent simulation steps (Phillips et al., 2014; Wang et al., 2015; Kim et al., 2020b).

#### 2.3.3 Absolute binding free energy calculation

As outlined in Kim et al.'s work, binding free energy calculations were conducted via the free energy perturbation method. This approach determined the binding free energy by subtracting the ligand’s free energy from that of the complex. These values were derived from simulations obtained from NAMD 3.0. Simulation inputs were generated from Charmm-GUI, with FEP simulations conducted over 10 ns, after a 5 ns equilibration in the NPT ensemble at standard conditions. Final energy values were taken from the last 5 ns, with VMD used for trajectory analysis. Ngo et al.'s benchmark study confirmed FEP’s accuracy in predicting M^pro^ inhibitors (Kim et al., 2020b; Ngo et al., 2021).

#### 2.3.4 Steered molecular dynamics simulation

SMD experiments were performed using NAMD, with force profiles across different compounds compared at a constant velocity of 0.025 Å/ps and a spring constant of 7 kcal/mol/Å^2. Optimal pulling rates were determined to balance resolution and time efficiency. Simulations lasted 1.3 ns, adequately capturing ligands unbinding. Results were averaged from three independent runs, with the most favorable poses serving as simulation starting points. Lys-67, Glu-89, and ASP-186 were set to be the anchoring residues (Alhadrami et al., 2022).

### 2.4 Linear regression analysis

The inhibitory concentration (IC_50_) values, expressed in micromolar (μM), were obtained from the Pim-1 assay. These values were converted to their corresponding negative logarithmic scale (pIC_50_) to facilitate a linear comparison using the following formula:

$$\text{pIC}50=-\log10\;\left(\text{IC}50\text{ in }M\right)$$

Where IC_50_ values were first converted from μM to molar (M) units before logarithmic transformation. Pulling force values, measured in pN, were sourced from the SMD experiments.

A linear regression analysis was conducted to explore the relationship between the pulling force and pIC_50_ values. The analysis was performed using the linregress function from the SciPy library in Python, which computed a least-squares regression for two sets of measurements. The model assumed a linear relationship of the form:

pIC_50_ = intercept + (slope × pulling force)

Where the slope represents the change in pIC_50_ with respect to the pulling force, and the intercept is the predicted pIC_50_ when the pulling force is zero.

The strength and direction of the relationship were quantified by the correlation coefficient (r-value), and the statistical significance was determined by the *p*-value. Additionally, the standard errors of the slope and intercept provided estimates of the variability in these parameters (Montgomery et al., 2021).

## 3 Results

**FIGURE 3:**
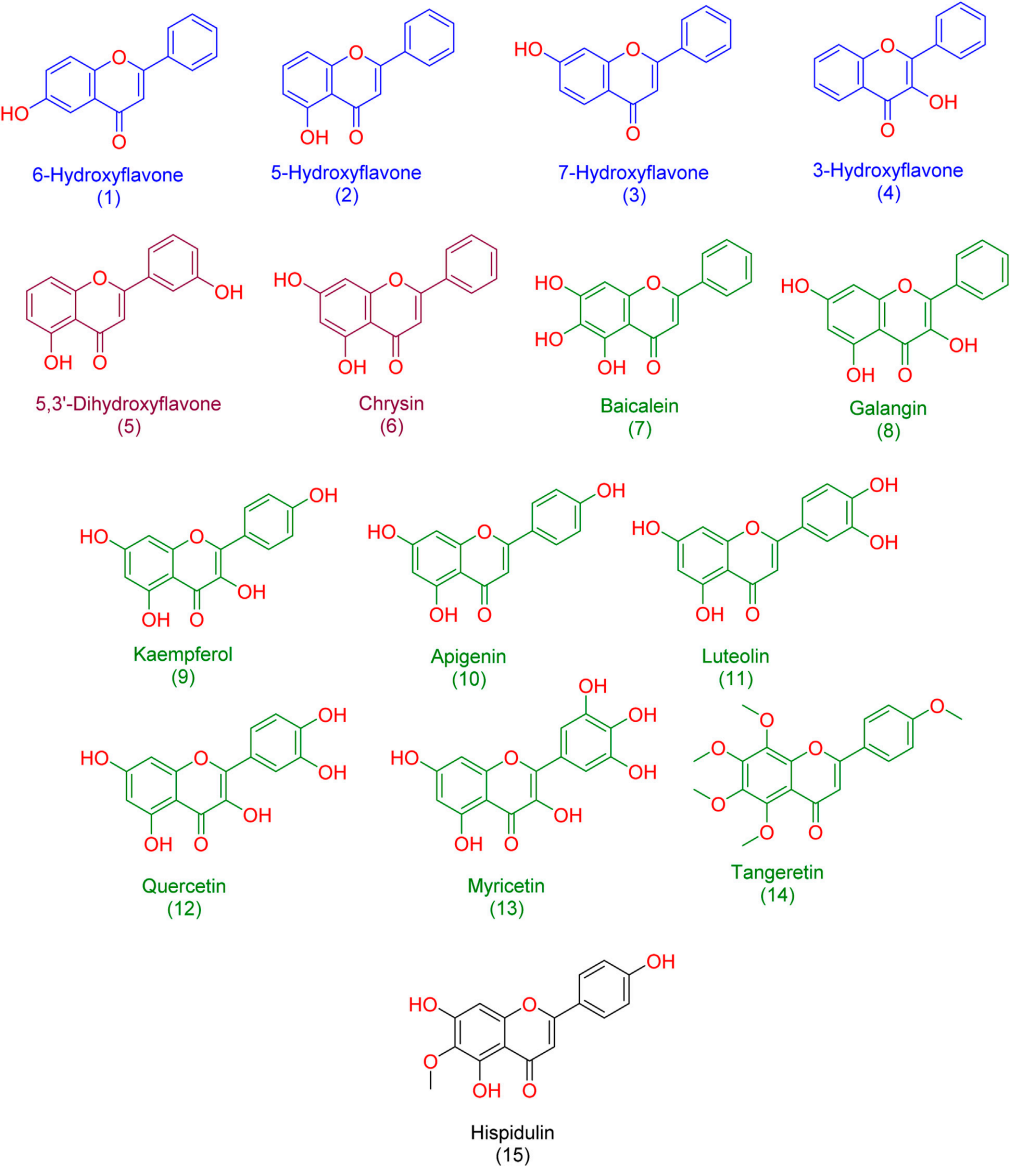
Structures of the studied flavonoids in the present investigation. one to four represented monohydroxylated flavonoids; five and six represented dehydroxylated flavonoids; 7-15 represented polyhydroxylated flavonoids.

In our study, we delved into the biological efficacy of flavonoids against Pim-1 kinase by merging docking methodologies with steered molecular dynamics (SMD) simulations. This approach sheds light on the intricate dynamics within the ligand-protein complex. Our initial step involved docking analyses of all flavonoid structures (1-15; Figure 3) to probe their interactions with Pim-1 kinase.

### 3.1 Docking-based study

**FIGURE 4:**
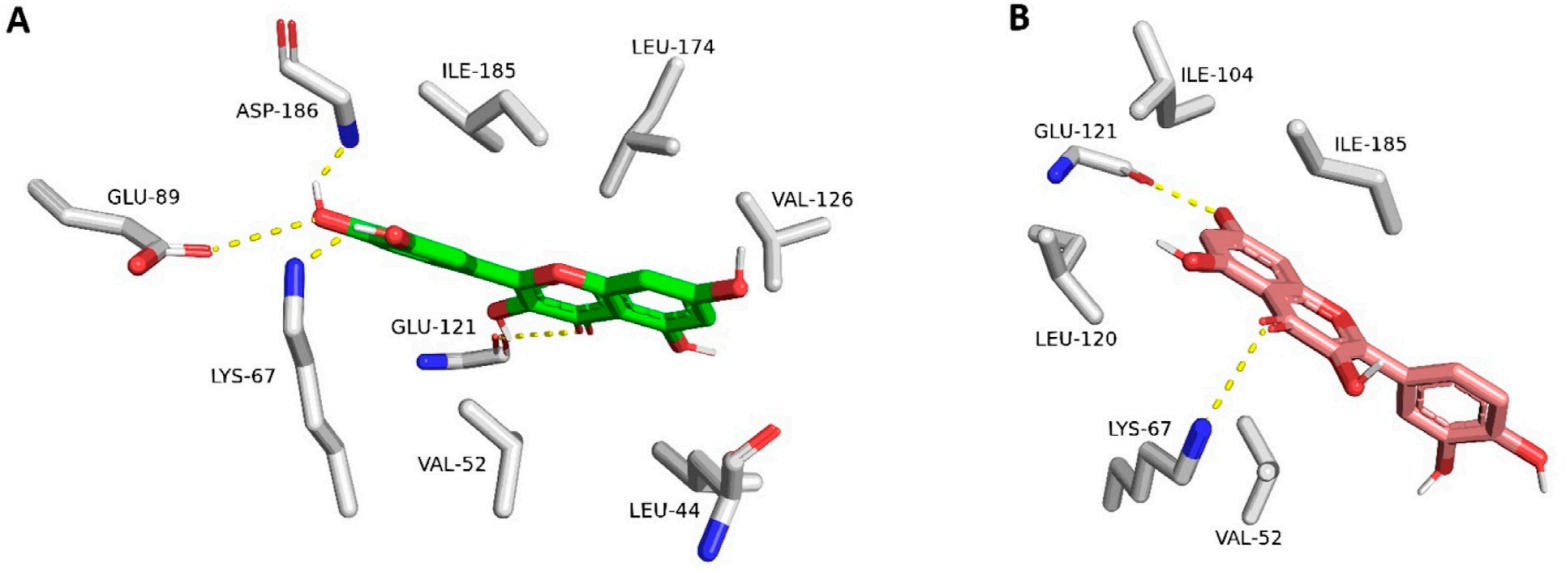
Quercetin interacting with *Pim-1 kinase’s* active site (PDB ID: 4LMU). **(A)** The best-scoring docking pose (Mode A; green structure). **(B)** The most populated docking pose (Mode B; brick red structure). The dotted yellow lines stand for H-bond interactions.

The resulting docking poses (10 poses for each docked structure) were in only two distinct orientations (Modes A and B, Figure 4). Mode A was always the best-scoring pose, while Mode B was the most populating pose among the resulting 10 poses for each docked structure. Mode A for docked quercetin was identical to the reported co-crystallized one (RMSD = 0.79 Å) (Holder et al., 2007; Parker et al., 2014).

Structures 13-15 were exceptions because their binding poses were all the same orientation that differed from either Mode A or Mode B. Such a result could be attributed to the steric hindrance of the polyhydroxy or polymethoxy substitutions (compounds 13 and 14, respectively). The best-scoring docking pose of compound (15) was identical to that of the reported one for hispidulin (RMSD = 0.91 Å) (Chao et al., 2015).

### 3.2 Molecular dynamics-based study

**FIGURE 5:**
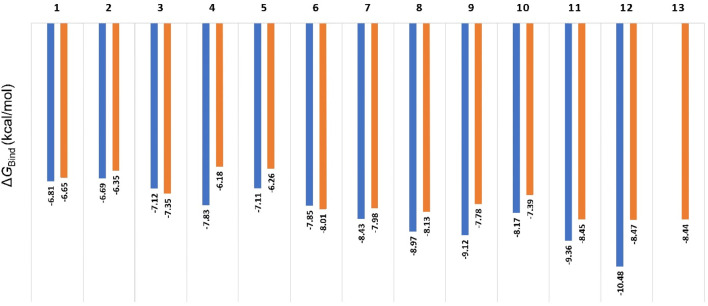
Calculated ΔG_Bind_ values for compounds 1-13 inside Pim-1 kinase’s active site in their two modes (A and B; blue and orange bars, respectively). The best scoring pose of each mode was selected for the calculations. All the resulting docking poses for Compound 13 were related to the binding mode B, so there was no value for Mode A regarding compound 13. Compounds 14 and 15 got completely different binding poses due to the steric hindrance of their methoxy groups, so they were excluded from the comparison.

In order to discern between the best scoring pose (Mode A) and the most populated one (Mode B), we first ran 50 ns-long for each resulting pose to calculate their binding free energies (Δ*G*_Bind_). As shown in Figure 5, the calculated values were convergent with slight preferences toward binding mode A, except for compounds three and 6. The calculated values ranged from −6.18 to −10.48 kcal/mol (i.e., quercetin in its binding Mode A), which indicated a good affinity towards the enzyme’s active site. In addition, the Δ*G*_Bind_ values for Mode A and B showed very good negative correlation coefficient (−0.858 and −0.857 for Mode A and B, respectively; using Person method) with the experimental IC_50_ values (Used as pIC_50_ the calculations). However, Δ*G*_Bind_ calculation was poor in discriminating between active and inactive binding poses (Figure 5).

### 3.3 SMD study

**FIGURE 6:**
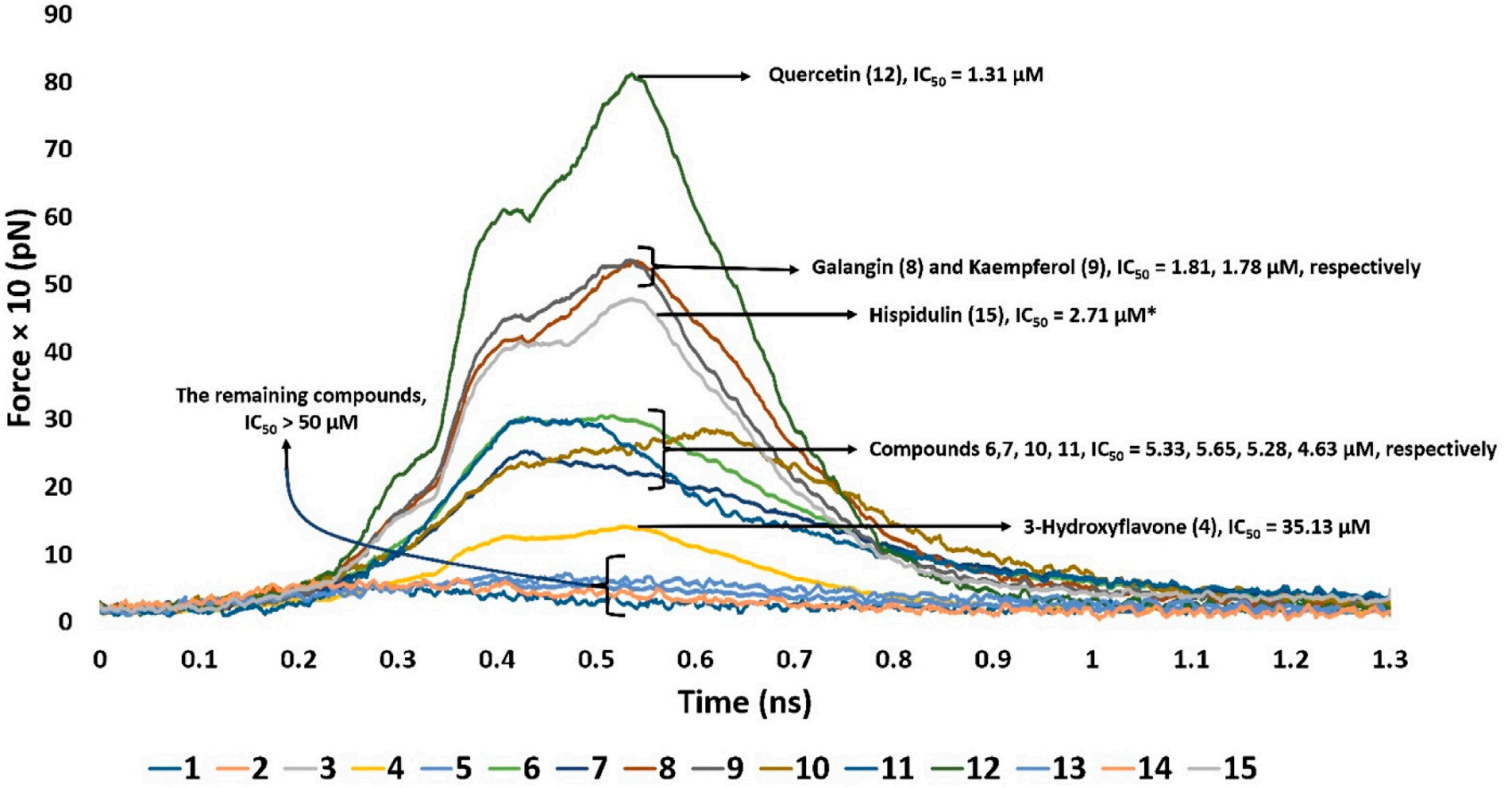
Comparison of the pulling force profiles and their Pim-1 kinase inhibitory activities (expressed in IC_50_) of different flavonoid structures. Compounds 6-12 were the most active derivatives, with more than 200 pN pulling forces and IC_50_ values lower than 6 µM. Quercetin (12) was the most potent inhibitor and recorded the highest puling force profile (≈820 pN). On the other hand, 3-hydroxy flavone (4) had the least inhibitory activity and pulling force (≈109 pN). Compounds eight and nine were clustered around ≈513 pN and got IC_50_ of 1.81 and 1.78 µM, respectively. Compounds 6, 7, 10, and 11 were clustered around ≈240–280 pN with IC_50_ values between 4.5 and 5.6 µM. Hispidulin (15) got a pulling force profile of ≈489 pN, however its binding pose did not align with either Mode A or B.

Second, we ran SMD experiments for each resulting pose. Each experiment was conducted twice. The resulting pulling force profiles clearly indicated that Mode B was unlikely the correct binding pose, where the average pulling force of all structures that represented this pose did not exceed 100 pN. On the other hand, binding poses that represented Mode A showed varying pulling force profiles (Figure 6). Interestingly, the resulting pulling force profiles were in perfect accordance with the *in vitro* enzyme inhibition results (Figure 6).

Derivatives 6–12 displayed the highest Pim-1 kinase inhibitory activity, exhibiting pulling forces exceeding 200 pN and IC_50_ values below 6 µM. Among them, quercetin (12) emerged as the most effective inhibitor, achieving the greatest pulling force of approximately 820 pN. Conversely, 3-hydroxy flavone (4) showed the lowest inhibitory capacity and pulling force, around 109 pN. These observations might be attributed to the number of H-bonds formed between the ligand and the active site’s residues, where quercetin (12) established the highest number of H-bonds (8-H-bonds). Moreover, the H-bonding with GLU-121 appeared crucial for stable binding (i.e., higher pulling force) and hence the enzyme’s inhibitory activity.

Compounds eight and nine were grouped together with pulling forces near 513 pN and respective IC_50_ values of 1.81 µM and 1.78 µM, respectively. The group comprising compounds 6, 7, 10, and 11 demonstrated pulling forces in the range of approximately 240–280 pN and IC_50_ values spanning 4.5–5.6 µM. Hispidulin (15), while achieving a pulling force of about 489 pN, did not conform to either binding Mode A or B in its interaction. However, its high pulling force (≈489 pN) was also correlated with good inhibitory activity.

### 3.4 Linear regression analysis of the SMD and the *in vitro* inhibition results

**FIGURE 7:**
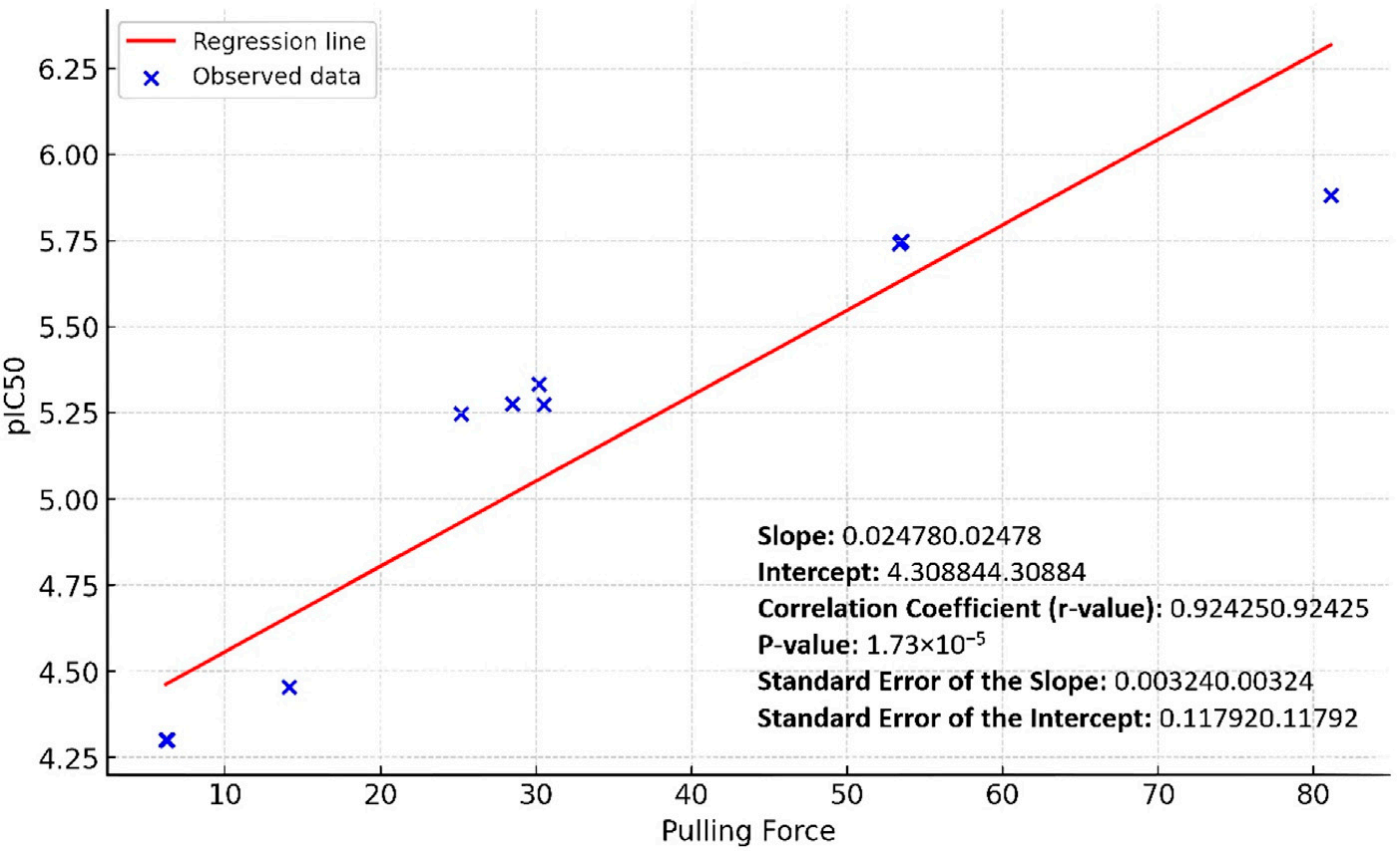
Linear regression curve of the Pim-1 kinase pIC_50_ values against the SMD pulling force values.

By applying a linear regression analysis between the pulling forces and the corresponding pIC_50_ values of the studied derivatives, we were able to establish a positive predictive correlation between the pulling force and the Pim-1 kinase inhibitory activity. As shown in Figure 7, the positive slope indicated a positive relationship between the pulling force and the pIC_50_ values: as the pulling force increases, so does the pIC_50_. The correlation coefficient (Person method) of approximately 0.92 suggested a strong positive linear relationship between the two variables (Figure 7). The *p*-value was very small (1.73 × 10^−5^), which indicated that the relationship observed was statistically significant, and the likelihood that the relationship between the pulling force and pIC_50_ values due to chance was very low.

The standard errors of the slope and intercept measured the variability of these estimates from the sample data. The relatively low values (0.00324 and 0.11792, respectively) suggested that the estimates were precise. Accordingly, the linear regression analysis showed that the pulling force significantly predicted the pIC_50_ values, with a solid and statistically significant positive linear relationship.

### 3.5 Investigating the dynamic interactions of potent inhibitors

**FIGURE 8:**
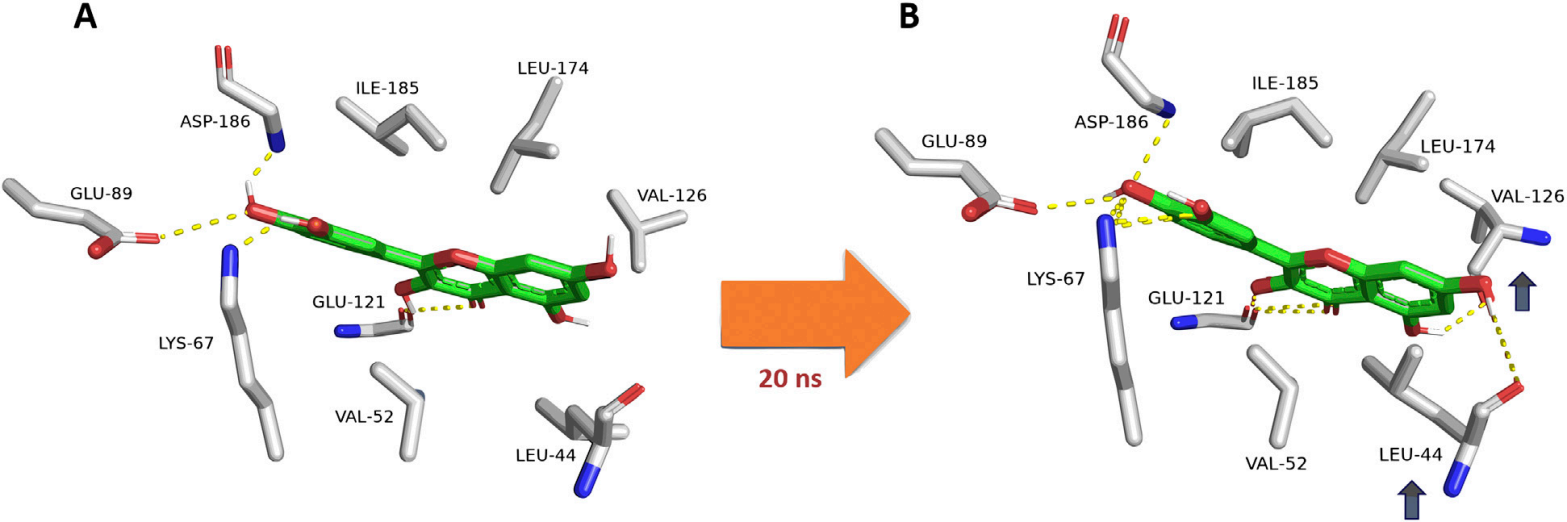
Dynamic binding mode of quercetin (12) inside the Pim-1 kinase active site during its MD simulation. Quercetin (12) was chosen as a model for all derivatives containing hydroxyl group at C-3. **(A)** The initial binding mode of quercetin at 0 ns (Mode A binding pose). **(B)** After ≈20ns, Leu-44 and Val-126 became closer to C-5 and C-7 hydroxyl groups, respectively, where they established two stable H-bonds lasting to the end of 200-ns long MD simulation. In addition, both residues were able to sandwich the structure’s ring A via hydrophobic interactions. This observation did not occur with C-3 dehydroxylated derivatives, where its interactions with Glu-121 were transient.

Upon further analysis of the dynamic interactions of the active compounds over 200 ns-long MD simulations, we were able to trace an exciting observation, as depicted in Figure 8, quercetin (12), serving as a representative for all derivatives containing a hydroxyl group at the C-3 position, exhibited a dynamic interaction pattern. At the start of the simulation (0 ns), quercetin was positioned in a specific manner (Mode A). As the simulation progressed, around the 20-ns mark, notable changes were observed in the interaction between quercetin and two amino acids, Leu-44 and Val-126. These amino acids moved closer to the C-5 and C-7 hydroxyl groups of quercetin, respectively, forming two stable hydrogen bonds that persisted throughout the 200-ns duration of the simulation. Additionally, these amino acids engaged in hydrophobic interactions, effectively sandwiching ring A of quercetin’s structure. This specific interaction pattern contrasted with derivatives lacking the hydroxyl group at C-3, where the interaction with another amino acid, Glu-121, was only temporary and not sustained over the simulation period. Hence, the approach of both Leu-44 and Val-126 towards quercetin’s ring A added more hindrance to its pulling outside the enzyme’s active site and, in turn, had more affinity to bind with it.

This crucial observation highlighted the significance of specific molecular interactions in the binding dynamics of small molecules with enzymes. In this case, the presence or absence of specific functional groups, like the hydroxyl group at the C-3 position, could dramatically influence how a molecule interacts with its target, which is crucial for understanding drug design and enzyme function. This example underscores the complexity and specificity of molecular interactions in biological systems, a fascinating aspect of biochemistry and molecular biology.

**FIGURE 9:**
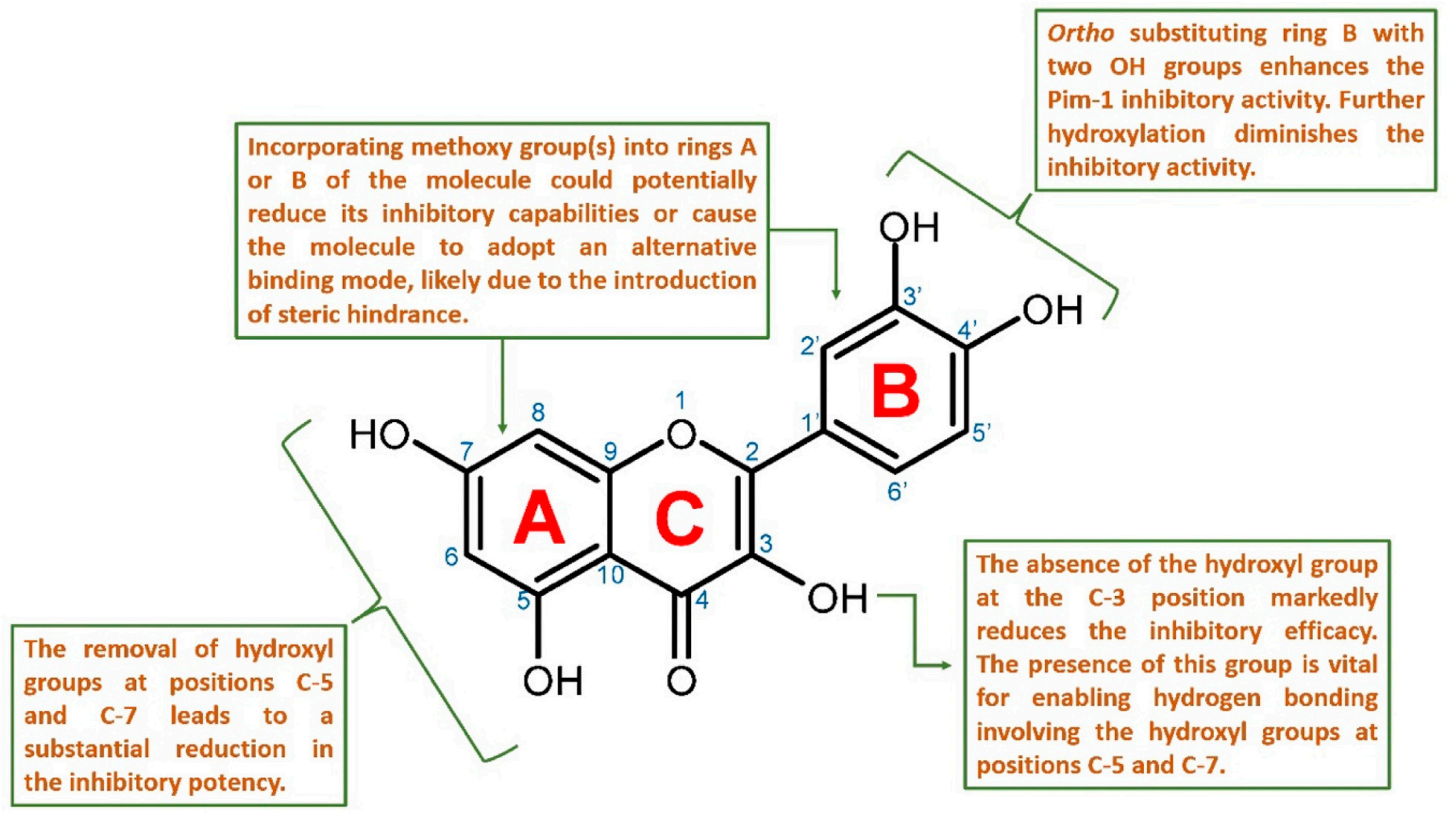
Proposed structure-activity relationship for the studied flavonoids as Pim-1 kinase inhibitors.

According to the previous findings, we could conclude a primary structure-activity relationship for this class of flavonoids as Pim-1 kinase inhibitors (Figure 9). There was a delicate interplay between molecular modifications and inhibitory potential. Flavonoids, a diverse class of polyphenolic compounds, exhibit their inhibitory activity through interactions within the kinase’s active site, and subtle changes to their structure can significantly alter their efficacy.

Key hydroxyl groups on the flavonoid scaffold played a crucial role in this interaction. For instance, the *ortho*-positioned hydroxyl groups on ring B were particularly important; their presence had been shown to enhance the inhibitory activity against Pim-1 kinase (e.g., quercetin). This suggested that these hydroxyl functionalities were critical for establishing strong hydrogen bonds or electrostatic interactions with essential amino acids within the active site, stabilizing the flavonoid and disrupting the kinase’s function. Further hydroxylation at ring B diminished the inhibitory activity (e.g., myricetin).

Conversely, the addition of methoxy groups to any part of the flavonoid rings might impede inhibitory activity. Such additions could lead to steric hindrance; a physical obstruction that prevented the flavonoid from fitting into the active site properly. This change could prompt the flavonoid to adopt a different binding mode that is less effective or entirely ineffective at inhibiting the kinase.

Moreover, the presence of hydroxyl groups at positions C-5 and C-7 was also significant for maintaining the inhibitory prowess of flavonoids. The absence of these groups had been correlated with a marked decrease in inhibitory activity, likely due to the loss of additional hydrogen bonding that contributed to the proper orientation and binding of the flavonoid within the active site.

Finally, the hydroxyl group at the C-3 position was described as crucial for the inhibition of Pim-1 kinase. This group likely interacted directly with Glu-121 in the active site, forming a critical part of the interaction network that allowed the flavonoid structure to strongly fit inside the active site and subsequently exert its inhibitory effect. It also allowed the inhibitor to form additional stable H-bonds via either C-5 or C-7 hydroxyl groups or both with Leu-44 and Val-126, respectively.

In summary, the SAR of flavonoids as Pim-1 kinase inhibitors (Figure 9) emphasized the importance of specific hydroxyl groups for optimal binding and inhibitory function. Modifications that preserve these hydroxyl groups and maintain the spatial conformation of the flavonoid are likely to be beneficial. At the same time, those that introduce steric clashes or remove essential hydrogen bonding capabilities are detrimental to the inhibitory activity. This knowledge provides a valuable framework for the design of new flavonoid-based inhibitors with enhanced potency and specificity for Pim-1 kinase.

### 3.6 Exploring the impact of inhibitors on the overall dynamics of Pim-1 kinase

To get some insight into how the investigated flavonoid Pim-1 kinase inhibitors might influence the global dynamics of the enzyme, we analyzed the resulting MD dynamics data in terms of eigenvectors. Such data are usually huge and diverse; hence, principal component analysis (PCA) of eigenvectors is the best way to describe these data in the simplest possible way and highlight the investigated protein’s structure dynamic behaviors. PCA is a statistical technique widely used in data analysis and machine learning to reduce the dimensionality of large datasets while preserving as much of the data’s variation as possible. In the context of molecular dynamics (MD) simulations and structural biology, PCA is applied to analyze the complex movements and conformations of molecules, such as enzymes, under various conditions (e.g., free, unliganded enzymes versus inhibitor-bound states).

Researchers can design inhibitors that more effectively modulate enzyme activity by understanding the differences in the principal components between free and inhibitor-bound states. This involves targeting the specific conformations or dynamics critical for the enzyme’s function, leading to the development of drugs with improved specificity and reduced side effects.

**FIGURE 10:**
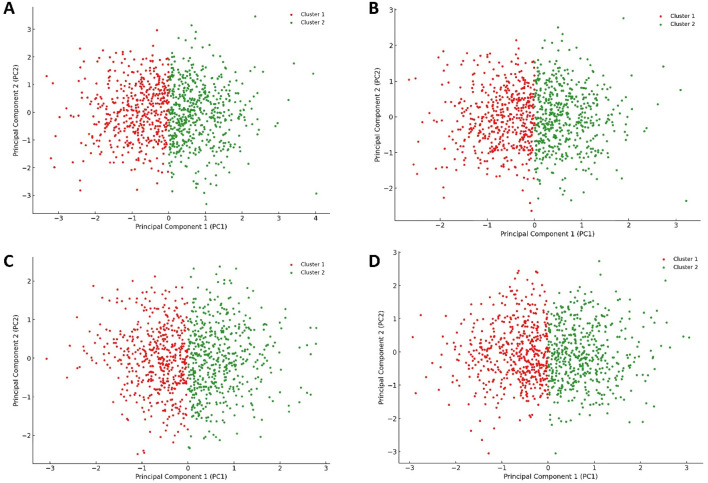
Projections of human Pim-1 kinase trajectories on the first two PCA eigenvectors of the MD trajectories. **(A)** represents the PCA plot of the unliganded Pim-1 kinase; **(B)** represents the PCA plot of Pim-1 kinase bound to quercetin; **(C)** represents the PCA plot of Pim-1 kinase bound to galangin; **(D)** represents the PCA plot of Pim-1 kinase bound to kaempferol. These bound compounds represent the flavonoids that potently inhabited Pim-1 kinase.

When comparing the PCA plot of the unliganded Pim-1 kinase structure (Figure 10A) with that representing Pim-1 kinase bound to potent inhibitors like quercetin, galangin, and kaempferol (Figures 10B–D, respectively), we could extract valuable insights into how the binding of an inhibitor influences the conformational landscape and dynamics of Pim-1 kinase.

The unliganded PIm-1 kinase plot showed a distribution of configurations that Pim-1 kinase adopted in its unliganded state, with the identification of two main clusters (Figure 10A). These clusters likely represented different conformational states or dynamical behaviors the kinase naturally explored in the absence of a ligand. The spread and distribution of these clusters could suggest a relatively flexible conformational landscape, allowing Pim-1 kinase to transition between various states, potentially relevant to its catalytic activity or regulatory mechanisms.

In contrast, the plots representing Pim-1 bound potent inhibitors (e.g., quercetin, galangin, and kaempferol) (Figures 10B–D) showed different distribution patterns. Assuming the inhibitor binding induced a more constrained dynamical behavior, we might observe a reduced spread in the data points or the formation of new clusters, suggesting that these inhibitors stabilized specific conformational states of Pim-1 kinase. The presence of such inhibitors bound to Pim-1 kinase might restrict the conformational freedom of the enzyme, as evidenced by potentially tighter clusters or a reduced spread of configurations (Figures 10B–D). This suggested that these inhibitors effectively limited the kinase’s ability to explore its entire conformational landscape, which is a desired effect in the context of inhibition.

**FIGURE 11:**
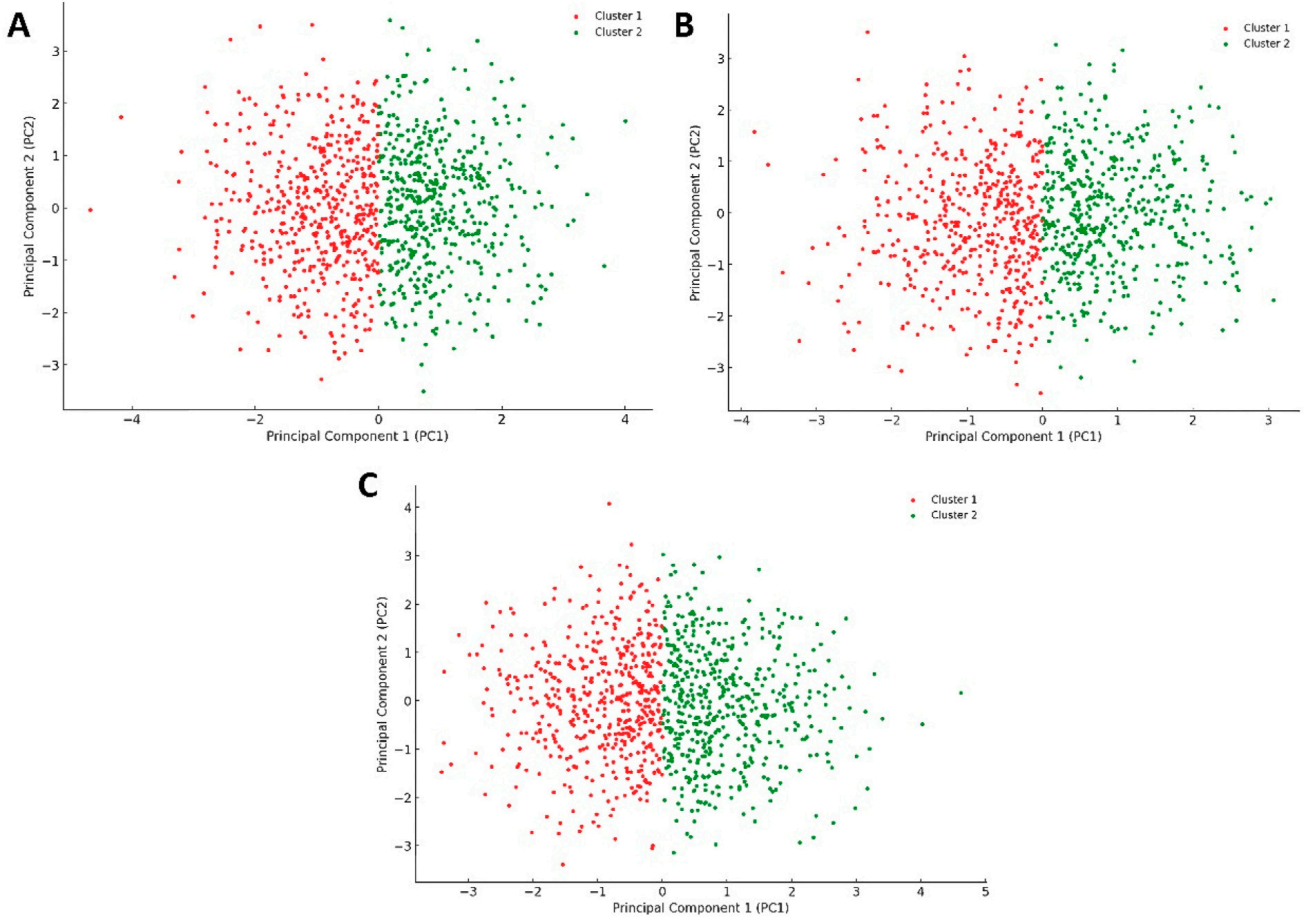
Projections of human Pim-1 kinase trajectories on the first two PCA eigenvectors of the MD trajectories. **(A)** represents the PCA plot of Pim-1 kinase bound to 5-hydroxyflavone; **(B)** represents the PCA plot of Pim-1 kinase bound to 6-hydroxyflavone; **(C)** represents the PCA plot of Pim-1 kinase bound to 7-ydroxyflavone. These bound compounds represent the flavonoids that could not inhibit Pim-1 kinase activity.

Conversely, the plots for Pim-1 kinase bound to weak inhibitors (e.g., 6-hydroxyflavone, 5-hydroxyflavone, 7-hydroxyflavone; Figures 11A–C) showed a return towards increased variance and broader distribution of configurations, akin to the unliganded state (Figure 10A) but with some level of constraint. These plots suggested that weak inhibitors had a less pronounced effect on the enzyme’s conformational dynamics. They might induce minor conformational preferences or slightly limit the enzyme’s flexibility without significantly altering its overall conformational landscape. The similarity among these plots indicated a consistent pattern of weak inhibition, where the enzyme retained a considerable degree of dynamical freedom, suggesting that these inhibitors did not effectively block the enzyme’s active site or essential conformational changes.

**FIGURE 12:**
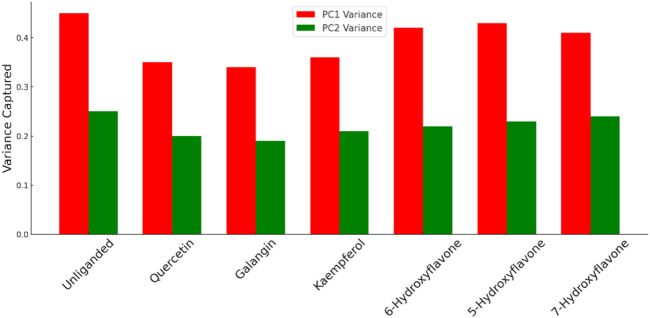
Comparison between the variance of PC1 and PC2 for Pim-1 dynamics in both the unliganded and inhibitor-bound states. Higher variance indicates higher structural dynamic freedom and flexibility and *vice versa*. Less structural flexibility is always associated with enzyme inhibition.

The previous PCA analysis could be further concluded in Figure 12. The Figure visually summarized the enzyme’s transition from a state of high conformational diversity in its unliganded form to more restricted states upon binding with inhibitor. The degree of restriction varied with the inhibitor’s potency, offering a clear visual representation of how different inhibitors could influence the enzyme’s structural and functional landscape.

The “distance from the center” in the context of our PCA plots referred to the Euclidean distance of each data point (representing the enzyme’s conformation at a specific time) from the centroid of the data distribution in the PCA-transformed space. This metric served as indicator of conformational diversity and dispersion within the PCA space, reflecting how broadly the enzyme explored its conformational landscape under various conditions.

A larger average distance from the center suggests that the enzyme samples a wider range of conformations, indicating greater conformational flexibility. This is crucial for the enzyme’s function, as different conformations can facilitate substrate binding, catalysis, and regulation. Hence, the impact of inhibitors on the enzyme’s conformational space is quantitatively reflected by changes in this distance. Potent inhibitors tend to reduce the average distance from the center, signifying a restriction of conformational flexibility as the enzyme is stabilized in fewer specific conformations. Conversely, weak inhibitors lead to a smaller reduction in this distance, implying that they exert a less pronounced effect on the enzyme’s conformational dynamics, allowing it to retain more of its inherent flexibility.

**FIGURE 13:**
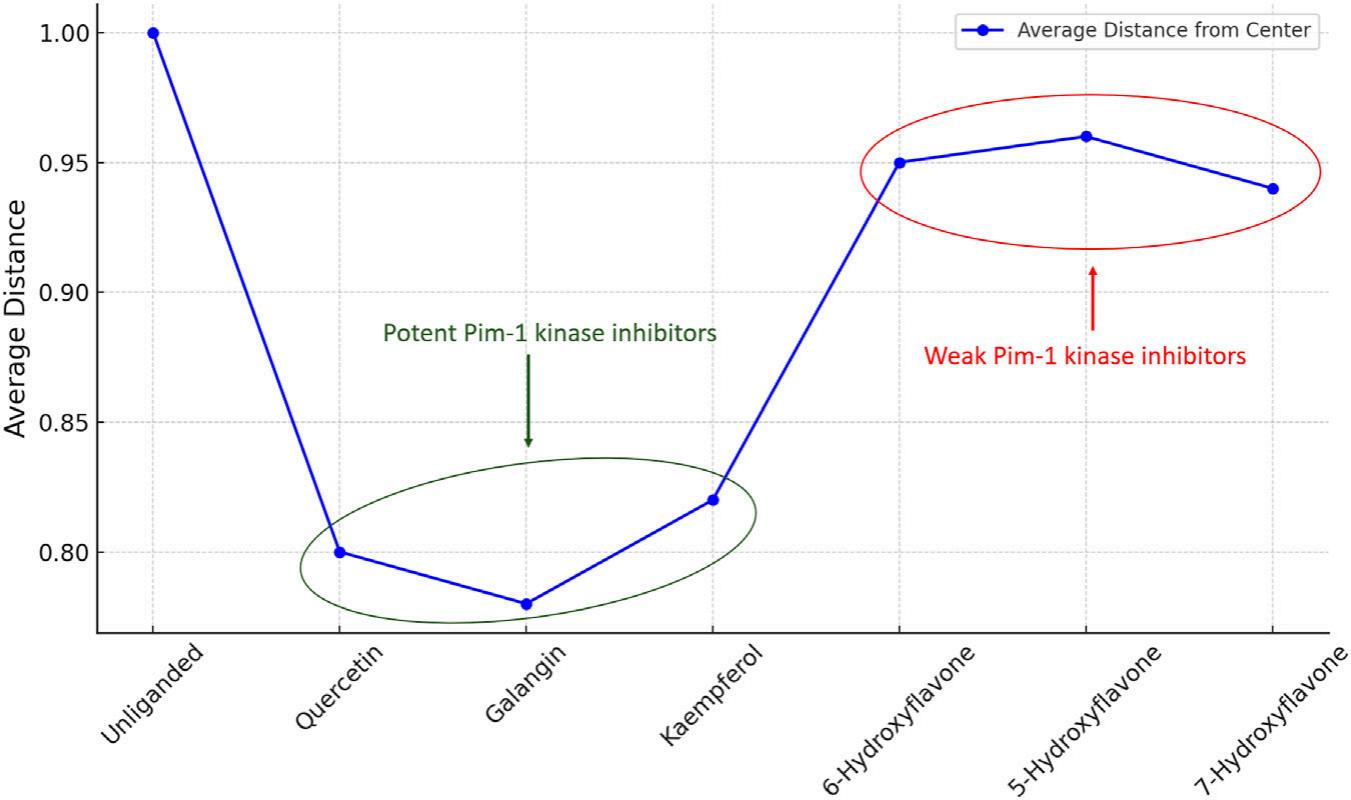
Distance from the center values of Pim-1 kinase eigenvectors data distribution in the PCA-transformed space in the unliganded and inhibitors-bound states.

The analysis of this metric (i.e., distance from the center) across unliganded and inhibitor-bound states (Figure 13) revealed the mechanistic basis by which inhibitors modulated enzyme activity, either by significantly restricting conformational exploration in the case of strong inhibitors or by subtly influencing it in the case of weak inhibitors.

This quantitative aspect adds depth to our understanding of the structural and functional implications of inhibitor binding, emphasizing the critical role of conformational dynamics in enzyme regulation and drug design. By elucidating the effects of different inhibitors on the conformational landscape of Pim-1 kinase, this analysis provides valuable insights for developing targeted therapeutic strategies that leverage the dynamic nature of enzyme structures.

## 4 Discussion

The search for effective Pim-1 kinase inhibitors has led to the exploration of various compound classes, with flavonoids emerging as a promising category due to their natural origin and potential for high-affinity binding. Our study provides structural insights into the interaction of flavonoids with Pim-1 kinase, to be used for further research into their potential as kinase modulators.

Our results emphasized the critical role of specific hydroxyl groups on the flavonoid scaffold in Pim-1 kinase inhibition. The ortho-positioned hydroxyl groups on ring B, as seen in quercetin, enhance the inhibitory activity by forming stable hydrogen bonds and hydrophobic interactions within the active site, a finding that corroborates previous research (Holder et al., 2007; Parker et al., 2014). The potency of these interactions is so significant that the removal of these hydroxyl groups leads to a pronounced decrease in inhibitory activity, likely due to the diminished capacity for binding orientation and stabilization within the kinase’s active site (Lilly et al., 2004).

Additionally, our study supports the hypothesis that adding bulky functional groups, such as methoxy groups, may interfere with the inhibitory potential of flavonoids by introducing steric hindrance. This observation is consistent with previous findings (Chao et al., 2015) and highlights the importance of carefully managing the steric profile of flavonoid derivatives to avoid disrupting interactions within the active site of Pim-1 kinase (Drygin et al., 2012; Zhao et al., 2019; Alnabulsi and Al-Hurani, 2020).

Our linear regression analysis between the pulling forces obtained from steered molecular dynamics (SMD) simulations and the inhibitory activities measured by IC_50_ values revealed a positive correlation, suggesting that SMD could be a valuable predictive tool in the early stages of inhibitor design. However, it is important to note that binding affinity alone cannot directly predict *in vivo* efficacy or pharmacological activity, as these depend on additional factors such as bioavailability, metabolism, and target specificity.

The dynamic binding mode analysis over extended molecular dynamics simulations has led to the noteworthy observation that quercetin (12) displayed a binding adaptability that was likely contributory to its high inhibitory potential. The ability of Leu-44 and Val-126 to establish and maintain stable hydrogen bonds with quercetin’s hydroxyl groups indicated a binding resilience that may be absent in derivatives lacking these critical functional groups. Our findings suggested that the hydroxyl group at the C-3 position is not just contributory but essential for the inhibition of Pim-1 kinase, likely due to its role in anchoring the flavonoid within the active site through key interactions with residues such as Glu-121.

Further structural dynamics study of Pim-1 kinase in its unliganded and inhibitors-bound states provided insights on the complex interplay between enzyme dynamics and inhibitor binding, emphasizing the pivotal role of conformational flexibility in enzyme function and regulation. This understanding is crucial for advancing drug discovery efforts, particularly in designing inhibitors that can precisely modulate enzyme activity.

While these findings offer important structural insights, it is essential to recognize that the computational and *in vitro* approaches employed here have inherent limitations. The results presented in this study provide a framework for further research rather than definitive conclusions about the therapeutic potential of flavonoid-based Pim-1 kinase inhibitors. The ability of flavonoids like quercetin to modulate Pim-1 kinase underscores the broader interest in natural compounds as potential leads in early-stage cancer drug discovery. However, it is well-known that polyphenolic compounds, including flavonoids, often exhibit pan-assay interference effects (PAINS), limiting their specificity and making it difficult to extrapolate *in silico* findings to clinical applications (Bajorath, 2021; Bolz et al., 2021; Wang et al., 2023). Despite this limitation, flavonoids remain an important area of study due to their diverse biological properties and interactions with multiple cancer-related targets, making them strong candidates for use in combination therapies, where their biological activity could complement and enhance the effects of established cancer drugs while potentially reducing toxicity and side effects.

Future experimental studies, including *in vitro* assays and *in vivo* models, are required to validate the inhibitory effects observed and to assess the clinical relevance of flavonoids in cancer therapy.

In light of these results, our study highlights critical structural features of flavonoids, such as the hydroxyl groups at specific positions, that contribute to their binding interactions with Pim-1 kinase. These findings offer a starting point for designing flavonoid derivatives that may have improved binding characteristics. However, the development of flavonoids as Pim-1 kinase inhibitors will require a careful balance between maintaining beneficial interactions and avoiding steric hindrance, with additional validation needed to confirm their potential therapeutic value.

## 5 Conclusion

This study elucidates the inhibitory mechanisms of flavonoids against Pim-1 kinase, highlighting the integration of steered molecular dynamics (SMD) and molecular docking to identify and understand the binding dynamics of inhibitors. Quercetin was identified as the most effective inhibitor, displaying the highest pulling force of approximately 820 pN and correlating with its low IC_50_ value (1.31 µM), indicating strong inhibitory potency. This establishes quercetin as a viable candidate for targeted cancer therapy. Further analysis confirmed a predictive correlation between SMD pulling forces and experimental IC_50_ values, validating the use of SMD in the early stages of drug discovery. The study also noted that the placement of hydroxyl groups significantly enhances flavonoid binding efficacy, while methoxylation, which introduces steric hindrance, reduces it. These insights inform the design of new flavonoid derivatives, advocating for the preservation of crucial hydroxyl groups to avoid steric clashes in the kinase’s active site. In conclusion, the integration of computational techniques, such as SMD and molecular docking, provides valuable insights into the structural dynamics of inhibitor binding, but further experimental studies, including *in vitro* and *in vivo* research, are necessary to explore the therapeutic potential of flavonoid-based Pim-1 inhibitors. While flavonoids alone may face challenges in clinical development as primary cancer therapies, their potential as adjuvants to enhance the efficacy of established treatments warrants further investigation.

## Data Availability

The original contributions presented in the study are included in the article/supplementary material, further inquiries can be directed to the corresponding author.
